# Local, collaborative, stepped and personalised care management for older people with chronic diseases (LoChro): study protocol of a randomised comparative effectiveness trial

**DOI:** 10.1186/s12877-019-1088-0

**Published:** 2019-03-04

**Authors:** Fabian Frank, Frederike Bjerregaard, Jürgen Bengel, Eva Maria Bitzer, Bernhard Heimbach, Klaus Kaier, Jasmin Kiekert, Lena Krämer, Cornelia Kricheldorff, Katharina Laubner, Andy Maun, Gloria Metzner, Wilhelm Niebling, Claudia Salm, Sandra Schütter, Jochen Seufert, Erik Farin, Sebastian Voigt-Radloff

**Affiliations:** 1 Department of Psychiatry and Psychotherapy, Medical Center – University of Freiburg, Hauptstraße 5, 79104 Freiburg, Germany; 2 Department of Social Work, Protestant University of Applied Sciences Freiburg, 79114 Freiburg, Germany; 3 Center for Geriatric Medicine and Gerontology, Medical Center – University of Freiburg, Lehenerstraße 88, 79106 Freiburg, Germany; 4 Department of Rehabilitation Psychology and Psychotherapy, Institute of Psychology, Albert-Ludwig-University of Freiburg, Engelbergerstraße 41, 79085 Freiburg, Germany; 5 Department of Public Health and Health Education, University of Education Freiburg, Kunzenweg 21, 79117 Freiburg, Germany; 6 Institute for Medical Biometry and Statistics, Faculty of Medicine and Medical Center, University of Freiburg, Stefan-Meier-Straße 26, 79104 Freiburg, Germany; 7 Institute for Applied Research, Catholic University of Applied Sciences Freiburg, Karlstraße 63, 79104 Freiburg, Germany; 8 Division of Endocrinology and Diabetology, Department of Medicine II, Medical Center – University of Freiburg, Hugstetter Straße 55, 79106 Freiburg, Germany; 9 Department of Medicine, Division of General Practice, Medical Center – University of Freiburg, Elsässerstraße 2m, 79110 Freiburg, Germany; 10 Section of Health Care Research and Rehabilitation Research, Medical Center – University of Freiburg, Hugstetterstraße 49, 79106 Freiburg, Germany; 11 Institute of Evidence in Medicine, Medical Center – University of Freiburg, Breisacher Straße 153, 79110 Freiburg, Germany

**Keywords:** Collaborative care, Elderly, Chronic disease, Multimorbidity, Depression, Diabetes, Frailty

## Abstract

**Background:**

Multimorbid older adults suffering from a long-term health condition like depression, diabetes mellitus type 2, dementia or frailty are at high risk of losing their autonomy. Disability and multimorbidity in the older population are associated with social inequality and lead to soaring costs. Our local, collaborative, stepped and personalised care management for older people with chronic diseases (LoChro-Care) aims at improving outcomes for older multimorbid patients with chronic conditions whose social and medical care must be improved.

**Methods:**

The study will evaluate the effects of LoChro-Care on functional health, depressive symptoms and satisfaction with care, resource utilisation as well as health costs in older persons with long-term conditions. The trial will compare the effectiveness of LoChro-Care and usual care in a cross-sectoral setting from hospital to community care. We will recruit 606 older adults (65+) admitted to local hospital inpatient or outpatient departments who are at risk of loss of independence. Half of them will be randomised to receive the LoChro-Care intervention, comprising seven to 16 contacts with chronic care managers (CCM) within 12 months. The hypothesis that LoChro-Care will result in better patient-centred outcomes will be tested through mixed-method process and outcome evaluation and valid measures completed at baseline and at 12 and 18 months. Cost-effectiveness analyses from the healthcare perspective will include incremental cost-effectiveness ratios.

**Discussion:**

The trial will provide evidence about the effectiveness of local, collaborative, stepped and personalised care management for multimorbid patients with more than one functional impairment or chronic condition. Positive results will be a first step towards the implementation of a systematic cross-sectoral chronic care management to facilitate the appropriate use of available medical and nursing services and to enhance self-management of older people.

**Trial registration:**

German Clinical Trials Register (DRKS): DRKS00013904; Trial registration date: 02. February 2018.

## Background

In Western countries, 65% of people aged 65–84 years are living with at least two long-term health conditions, rising to 82% of those aged ≥85 years [1]. In Germany, 58% of female and 55% of male older persons aged ≥65 reported at least one chronic disease in 2012 [2]. Late life depression [3], diabetes mellitus type 2 [4, 5], dementia [6] and frailty [7] increase the risk of functional disability in daily living. As chronic diseases and functional decline are often interrelated, old and very old people with multimorbidity are at high risk of losing their autonomy [8, 9]. Disability and multimorbidity in the older population represent a substantial burden [10] and lead to soaring costs [11]. Indeed, in Germany, persons with multiple chronic diseases have twice as many general practitioner contacts per annum as people without multimorbidity (36.3 versus 15.9) [12]. The increasing prevalence of comorbid long-term conditions is associated with growing rates of preventable and expensive complications.

In Germany, medical and nursing services such as hospital discharge planning [13], counselling of patients and caregivers [14], disease management programs [15], geriatric medical centres [16] and case management for single diseases in primary care settings [17, 18] are well established. However, a systematic approach to cross-sectoral chronic care management is lacking. Such an approach should efficiently guide older people with multimorbidity through the field of fragmented medical and nursing services, and effectively enhance their self-management and their use of informal support networks.

A recent analysis of care delivery in long-term conditions strongly recommended efforts to support the concept of “activated” patients, i.e. patients who are competent in (1) enhanced self-management, (2) identifying and using informal support capacities, and (3) appropriately utilising formal regional health care services [19, 20].

Systematic reviews of randomised controlled trials have yielded mainly moderate evidence suggesting that complex interventions including care and case management are feasible and effective in older people with depression [21, 22], diabetes mellitus type 2 [23, 24], and dementia [25, 26], and those at risk of losing their independence at home [27, 28]. However, for patients with multimorbidity, there is only a limited body of evidence on health outcomes and the cost-saving potential of enhanced care coordination in cross-sectoral chronic care, as shown by systematic reviews [29–31], cost analyses [32, 33], and as confirmed by a current Cochrane review [34] and a recent JAMA editorial [35].

## Study aim and objectives

Older people with multimorbidity frequently experience insufficiently coordinated care. Therefore, the project aims to improve the coordination and performance of their cross-sectoral routine health care and address the evidence gap between mono-disease approaches and complex collaborative chronic care in old and very old people with multimorbidity. In this respect, the objective of the trial is to evaluate the effectiveness of local, collaborative, stepped and personalised care management for older people with chronic diseases (LoChro-Care). The programme is designed to improve functional health, depressive symptoms, satisfaction with care, and health resource utilisation.

We hypothesise the following improvements, to be tested in comparison with usual care: (1) LoChro-Care will result in better functional health as defined by greater independence in activities of daily living. (2) LoChro-Care will more effectively reduce depressive symptoms. (3) LoChro-Care will improve satisfaction with care. (4) LoChro-Care will lead to a more appropriate utilisation of health and nursing care services in terms of decreased emergency hospitalisations, reduced non-elective hospital days and nursing home admissions, more adequate use of informal and formal community services and enhanced disease self-management abilities, and will thus contribute to health care cost savings. (5) Positive LoChro-Care effects will occur directly after the 12-month intervention and will be maintained for at least an additional six months after the intervention.

## Methods/design

### Trial design/setting

**Fig. 1:**
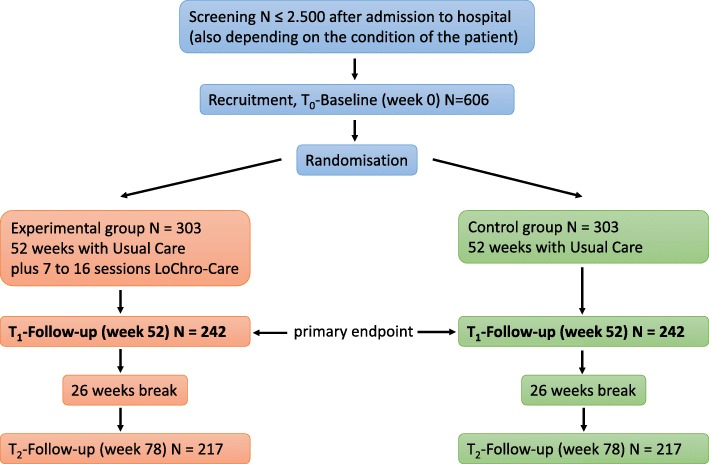
Trial Design

The present study will be conducted within the routine care context and is designed as a prospective, two-group usual care controlled trial with 1:1 randomisation on the participant level. Participants will be recruited from all for geriatric patients relevant inpatient and outpatient units of the Medical Center – University of Freiburg, especially the emergency unit. During the 18-month observation period, the chosen endpoints will be measured three times (at baseline, after 12 months and after 18 months). Participants in the intervention group will be supported by a chronic care manager (CCM) over a period of 12 months. The study will be implemented according to CONSORT guidelines (see Fig. 1).

LoChro is a cooperation between several departments from the Medical Center – University of Freiburg, the Albert-Ludwig-University of Freiburg, the Catholic University of Applied Sciences Freiburg, the University of Education Freiburg and the recruiting hospitals. There is extensive expertise in care management and research in the fields of geriatric, psychiatric, internal and general medicine as well as in gerontology, education, psychology, social work and health services research.

### Eligibility criteria

The target population comprises geriatric patients admitted to relevant inpatient and outpatient units of the Medical Center – University of Freiburg, aged ≥65 years and residing in or near Freiburg. Patient records will be checked for the presence of these inclusion criteria. Eligible patients will be screened using the “Identification of Seniors at Risk Screening Tool” (ISAR), which has proven to be sensitive in German emergency departments to identify older persons at risk of unplanned readmissions, institutionalisation and need for nursing care [36]. The ISAR tool takes less than five minutes to complete and includes only six simple questions, which are easy for patients or caregivers to answer. Thus, it is feasible to administer. ISAR scores range from 0 (no risk) to 6 (very high risk); persons with an ISAR score of 2 or more are considered to be frail multimorbid patients at risk, and will thus be included in the study. Exclusion criteria are: (1) terminal medical conditions, (2) lack of basic German-language skills or no German-speaking caregiver available, and (3) not residing in the region.

### Intervention group

LoChro-Care follows the ARIADNE principles for the management of multimorbidity [37]. These are: (1) a thorough assessment of interactions between the patient’s conditions, treatments, constitution, and context; (2) a prioritisation process of multiple health problems that takes into account the patient’s preferences, and (3) individualised management that utilises the best options for care and services. The personalised, stepped, cross-sectoral and collaborative LoChro-Care programme will be provided by a qualified nurse who specialises in health education or a social worker acting as CCM. They will be backed up by an interdisciplinary geriatric team, which will provide bi-monthly supervision and can be contacted in the case of urgent need for action. LoChro-Care will be initiated directly after the patients’ discharge from inpatient treatment and includes seven to 16 contacts within a 12-month period.

#### LoChro-care: Key agents and core elements

Novel aspects of LoChro-Care are reflected in the combination of the following approaches within one connected concept: (1) enhancing self-management based on stepped care plans delivered in plain language, which are understandable and foster easy communication between the patients or caregivers and service providers, (2) facilitating the appropriate use of informal support and the use of lay helpers if necessary, (3) supporting the CCM through an interdisciplinary geriatric team, (4) providing the CCM with selected online information on local geriatric services and (5) evaluating the stepped care plans and the spectrum of local geriatric services in its entirety to generate a best practice guideline for regional stepped collaborative care.

#### Implementation and time schedule of the intervention

**Fig. 2:**
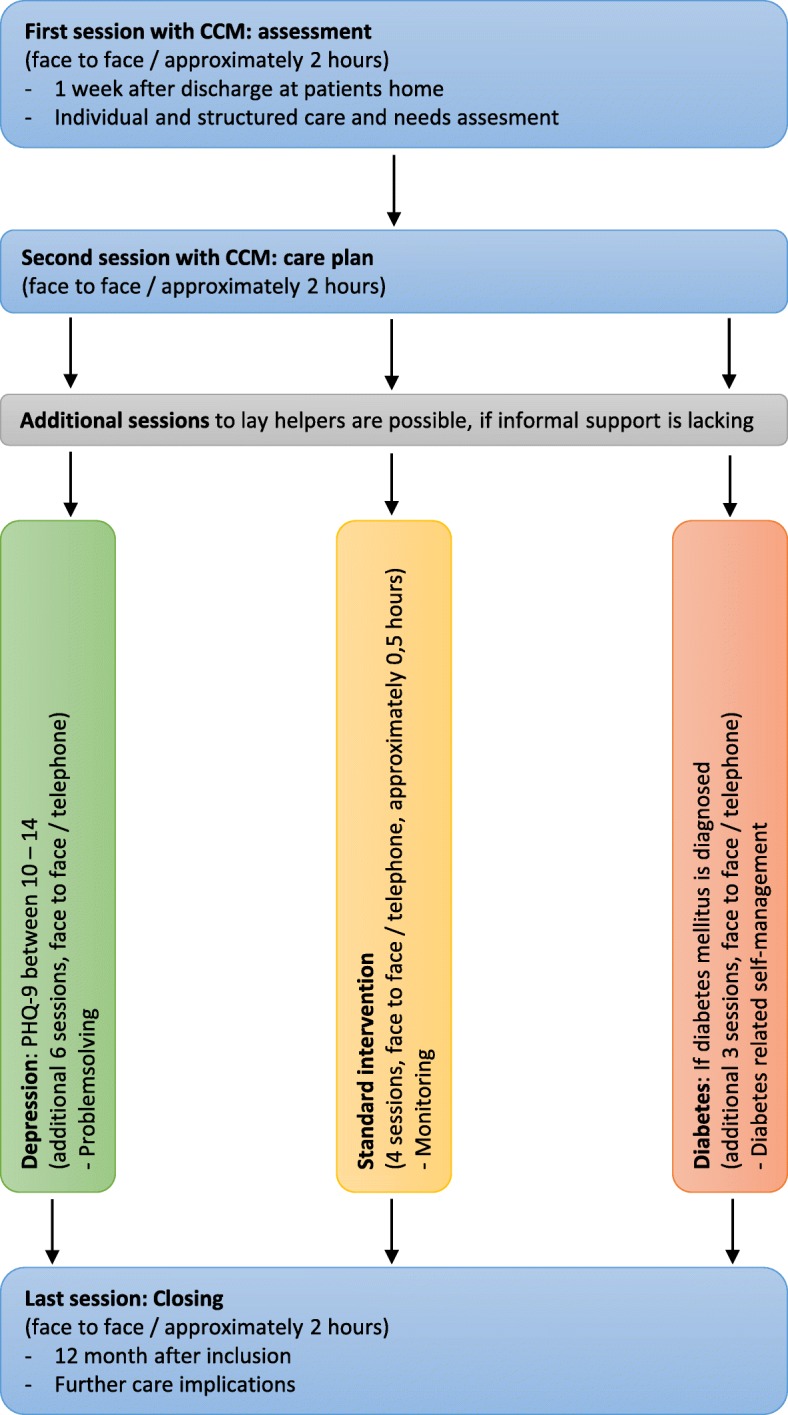
Sequence of intervention sessions provided by the CCM

After receiving the contact details, the CCM will arrange an appointment with the patient at the patient’s home for approximately one week after discharge. The assessment of the questionnaires summarised in Table 1 and individual interviews will form the basis to establish an individual care plan, which will be discussed and decided upon with the patient within two weeks after the first contact. The individual care plan consists of one or more of the following steps: (1) If the existing support by the general practitioner and family or other informal caregivers is sufficient, the CCM will compile, explain and deliver an individualised care plan including information about enhanced self-management and about geriatric outpatient services which are relevant and accessible to the patient. The CCM can choose relevant information about regional geriatric services from an internal project database including links to profiles and contacts. The CCM will encourage the patient to communicate the care plan to all informal and formal care partners. (2) If the patient suffers from cognitive impairment, the CCM intervention will involve the primary caregiver. (3) If there is no primary caregiver and other informal support is lacking, the CCM will establish contact with lay helpers who can serve in a supporting role. (4) If contacts with a general practitioner have been too infrequent or non-existent, the CCM will help to establish this provider relationship with adequate frequency of contact. (5) If the medication list reveals uncertainties for the patient or no medication list exists, the CCM will encourage the patient to get in touch with his/her general practitioner to work on medication problems. (6) If further formal care is needed, the CCM will help to contact the general practitioner and to establish contact with local geriatric services. Examples of local services involved are nursing day care, outpatient services, geriatric outpatient rehabilitation, housing agencies or private practices for physiotherapy or occupational therapy as well as further psychosocial facilities. Four weeks after the second contact, all planned steps will be monitored and discussed with the patient at his/her home. If no personal contact is required, the next three contacts will take place through telephone calls, in which the CCM discusses possible needs for care plan changes with the patient. The final “closing” contact will take place 50/51 weeks after baseline at the patient’s home and will consist of reflections on achievements as well as a summary of recommendations for future care. Additional contacts to this standard intervention are possible in the case of depression or diabetes. For depression, six additional contacts (at home and via telephone) will take place, in which patients are trained in problem-solving techniques. This method has proven successful in numerous depression trials [38, 39]. For diabetes as a present condition, three additional contacts at the patient’s home or via telephone are possible. These contacts will include diabetes-related self-management issues. Details about the sequence of intervention sessions can be found in Fig. 2. To ensure treatment fidelity, the CCM will receive regular specialist counselling by a geriatrician and by a study team member of the University of Education.

**Table 1:** LoChro outcome measures, instruments, and times of assessment

Time of assessment (months)	T_0_ (0)	CCM-session 1	T_1_ (12)	T_2_ (18)
Primary outcome				
Functional health (WHODAS 2.0)	X		X	X
Depression (PHQ-9)	X		X	X
Secondary outcomes				
Satisfaction with care (PACIC)	X		X	X
Ressource utilisation (FIMA)	X		X	X
STOPP/START criteria	X		X	X
Health-related quality of life*	X		X	X
Satisfaction with life (Kurzskala L-1)	X		X	X
Intervention group only				
Daily Functioning (Barthel-Index)		X		
Daily Functioning (IADL)		X		
Cognition (MMSE)		X		
Mood (PHQ-9)		X		
Alcohol Use (Audit C)		X		
Mobility (SPPB)		X		
Medication Management*		X		
Individual Interview on patient’s preferences and care situation*		X		

AUDIT-C: Alcohol Use Disorders Identification Test-Consumption; Barthel-Index: daily functioning in older people; FIMA: Fragebogen zur Erhebung von Gesundheitsleistungen im Alter; IADL: Instrumental Activities of Daily Living; Kurzskala L-1: single item scale on satisfaction with life; MMSE: Mini-Mental-State-Examination; PACIC: Patient Assessment of Chronic Illness Care; PHQ-9: Patient Health Questionnaire; SPPB: Short Physical Performance Battery; Stopp/Start: Stopp/Start criteria for potentially inappropriate prescribing in older people; WHODAS 2.0: World Health Organization Disability Assessment Schedule 2.0; *: self-constructed items

### Control group

Patients in the control group will receive usual cross-sectoral care management for old and very old people with multimorbidity at hospital discharge, including a medical referral letter to the patient’s general practitioner and, if indicated, referral to inpatient or outpatient rehabilitation or to outpatient nursing services or nursing homes.

### Outcome measures and assessment

#### Primary and secondary outcome

We reviewed publications on patient-centred outcomes as outlined in the PCORI Methodology Report [40], drew on systematic reviews on core outcome sets as suggested by the international COMET Initiative [41] and analysed reported outcomes from clinical trials in patients with multimorbidity. Based on our analysis of this literature, we conclude that outcomes with the highest relevance for the affected patients are (1) functional health in terms of daily activities (including cognitive and physical functioning), (2) mood (including depression) and (3) satisfaction with care. From a health care perspective, (4) resource utilisation, health care costs and appropriateness of medication plans will be evaluated. Thus, our evaluation plan encompasses both the individual and the health care perspective. We will use instruments that are already established and validated in German populations: The primary outcomes functional health and depression will be assessed with the “WHO Disability Assessment Schedule 2.0, WHODAS” [42] and the “Patient Health Questionnaire, PHQ-9” [43]. The secondary outcomes satisfaction with care, resource utilisation and medication appropriateness will be assessed with the “Patient Assessment of Chronic Illness Care, PACIC” [44], the “Fragebogen zur Inanspruchnahme medizinischer und nicht-medizinischer Versorgungsleistungen im Alter, FIMA” (questionnaire on the use of medical and non-medical services in old age) [45] and the Stopp/Start criteria [46] (see Table 1). Following TOPICS-MDS [47], quality of life and satisfaction with life [48] will additionally be assessed, each by a single-item scale.

In the intervention group, further instruments will be applied (see Table 1). These instruments, as well as the individual CCM interviews, form the basis to develop the individualised and personalised care plans; they are not part of the primary evaluation plan but can be used for additional analysis.

#### Assessment

The chosen endpoints will be measured at baseline and at 12 and 18 months using valid and reliable outcome assessment instruments as described above. We chose feasible and brief patient-reported outcomes in order to evaluate the participants’ perspective and to motivate the patients to become involved through self-reflection and feedback. If necessary, proxy versions of particular/single assessment instruments for caregivers will be used to avoid missing data, e.g. in persons with severe dementia. For proxy assessment, we will only use those instruments which do not require introspection and can thus be rated by caregivers. Additionally, the medication regimens for all enrolled patients will be recorded at baseline and at both follow-up time points.

#### Sample size and power calculation

Based on dropout rates of about 20% in our previous studies and small effects of case and care management on functional health, we hypothesise a Standard Mean Difference of 0.3 to be detected. We calculated a sample size of 606 persons to be randomised at baseline and 242 patients in each arm to be assessed at 12 months (one-sided t-test, significance level 0.05, 95% power, computed for per-protocol analysis, assumption 20% dropout at month 12).

#### Additional evaluation

At the beginning of the intervention, we will randomly select three cases from the experimental group with needs for slight vs. moderate vs. complex care, and match them with three cases from the control group according to relevant baseline characteristics and baseline assessment scores (e.g. diagnoses, sex, age, functional health, depression). At the end of the intervention, blinded physicians will retrospectively carry out a full chart review for all six cases, based on information from the patients’ general practitioners, in order to compare the one-year periods of care management and to gain a deeper understanding of procedures and decisions of care and how enhanced coordination might change care management. For the three experimental cases, we will additionally video-record the CCM sessions 3 and 7 (monitoring and closing contacts). After the blinded physician has completed the review of these six cases, he/she will be “unblinded” and will additionally assess the videos and the CCM documentation of the three experimental cases. Later, during the dissemination phase, these videos will be used for the training and qualification of routine care CCMs.

In addition, three participants of the experimental group will be separately selected at random and asked about their perceived quality of care using the method of open in-depth interviews and qualitative content analysis.

#### Recruitment of participants

Based on the annual statistics of the emergency department of the Medical Center – University of Freiburg, the access to the target population is estimated as follows: 42,000 patients per year present at the emergency department; of these, 11,000 persons are aged 65 or older, of whom 2500 presented with chronic or geriatric diseases and were locally resident in 2015. We expect that a quarter of these patients (approx. 600) will have eligible ISAR scores and will provide informed consent and that about 20% will drop out before the 12-month follow up, resulting in approximately 480 patients. As external assessors, our experienced geriatric staff are familiar with routine care procedures and will ensure that study activities neither drain department resources nor hinder usual care processes. In summary, we are well positioned not only to ensure adequate recruitment, but also to overcome any concerns of staff regarding participation in the study and to avoid recruitment bias due to implicit resistance.

#### Assignment and randomisation

After obtaining written consent from participating patients, external assessors responsible for the screening will send contact details of the patient, relatives as well as the baseline assessment to the primary coordinating centre. There, the allocation of the patients to the study arms will be performed. Participating patients will receive a pseudonymised code and will then be assigned to the intervention or control group according to a computer-generated randomisation schedule by a study coordinator not involved in assessment or intervention procedures. Block randomisation will be used with no stratification. After assignment to the intervention or control group, the pseudonymised patient identifiers and the baseline assessment will be sent to the study centres responsible for the data management. The study site responsible for the implementation of the intervention will receive names and contact details of the patients in the intervention group in order to begin the intervention process.

#### Blinding

In both groups, patient outcomes will be collected by independent assessors to prevent the contamination of the CCM by patient assessment. Due to the pseudonymisation process, the statistician will analyse the primary and secondary outcome data without knowledge of the subjects’ allocation to the study arms. For additional qualitative analysis of CCM data in the experimental group, this kind of blinding is not possible. Due to the nature of the intervention, participants and CCM will not be blinded to the type of intervention.

### Statistical analyses

The statistician will perform an intention-to-treat and per-protocol analysis of the outcome assessment scores at baseline and 12- and 18-month follow-up (dependent variables), adjusting for baseline group differences (independent variables). The hypotheses claim superiority of the intervention group over the control group with respect to the two outcomes (functional health (WHODAS) and depression (PHQ-9)) at 12- and 18-month follow-up. As we will use a composite endpoint of the two primary outcomes (sum of z-score normed component measures), no adjustment of the significance level is necessary. Subgroup analysis will be conducted to reveal group differences in intervention effects between patients with serious versus slight health-related strain. Groups will be classified by an ISAR score of > = 3 versus < 3. Furthermore, secondary outcomes (satisfaction with care, resource utilisation, medication appropriateness) will be analysed. The assumption of ´missing at random` is assumed for the problem of missing data. The fully conditional specification method (an iterative Markov chain Monte Carlo method) will be applied for the imputation of missing data. Dropout analysis will be performed comparing all baseline and follow-up data of completers and dropouts.

In exploratory analyses of all intervention cases, structural equation models will be used, including variables of three dimensions: (1) patient baseline characteristics, (2) CCM treatment procedures as actually carried out and (3) pre-post differences on outcome assessment scores. Through these analyses, we intend to identify possible interactions between characteristics of patients and treatment performance and to deepen our understanding of how the treatment might work and for which patients it can work best.

Cost-effectiveness analyses of LoChro-Care will be carried out from the healthcare perspective. Therefore, the change-from-baseline scores of the WHODAS, PHQ-9 and PACIC will be analysed in relation to the costs of medical resource utilisation and the costs of the work of chronic care managers. Incremental cost-effectiveness ratios and corresponding confidence intervals will be estimated using seemingly unrelated regressions, a multivariate regression technique that accounts for the potential correlation between cost and outcome (score) measurements. Based on data from the FIMA instrument, standardised unit costs will be applied to quantify the costs of medical resource utilisation. With the aim of increasing the precision of the assessment, we will complement the FIMA questionnaire with a prospective diary method in a subsample. Over a 12-month period, patients will be requested to record their medical resource utilisation. The diary is designed as a table including the main aspects of medical costs. Cost-effectiveness analyses will be conducted for the whole sample as well as for the subsample with diary cost assessments in the intervention and control group. In addition, the costs of the work of chronic care managers will be determined. We will consider employers’ labour costs and costs for the administration and coordination of the chronic care managers (lump sum of 25% of personnel costs).

### Ethical considerations and safety

#### Good clinical practice

The treatment plan and intervention techniques are compliant with recommendations of current national and international clinical guidelines. Control group patients will receive full usual care management according to ethical and legal standards as established in Germany. Experimental group patients will receive an add-on coordination without any additional risk and without any restriction of the usual care management. There is no evidence suggesting that care management increases the risk of morbidity, mortality or functional decline. If adverse events occur during the trial, they will be documented by the CCM or, for patients in the control group, by the data management team. However, there is no indication from studies that older people with multimorbidity are exposed to a higher risk of adverse events when participating in chronic care management programmes. Furthermore, the LoChro-Care study is a sponsor-driven trial without any commercial interest of the involved researchers, clinical staff or participants, and a scientific and practice advisory board has been initiated. Given these facts, in our judgement, the scientific and practice advisory board will sufficiently ensure quality and there is no need for specific committees on data monitoring and safety. All study materials and procedures will (1) be discussed a priori with the patient representatives of our practice advisory board, (2) adapted according to their feedback, (3) piloted with eligible patients, (4) again adapted to patients’ needs and (5) finally approved by our practice and scientific advisory board. The ethical committee of the University of Freiburg has reviewed our proposal regarding conformity with current ethical standards for participants’ safety and confidentiality and has given formal ethical approval (no. 495–17, date: 19th December 2017). All relevant protocol modifications and amendments will be submitted to the responsible ethical review boards and will be reported within the scope of the publication of the trial findings. Written informed consent is mandatory for each patient for enrolment in the study.

### Data management

#### Data protection

All patient data collected throughout the study, including documentation of the intervention sessions, will be stored and analysed using identifiers (pseudonyms) instead of patient names to grant the highest possible protection of privacy. Only a small number of authorised members of the leading investigating centre, the recruitment centre and the centre responsible for the intervention (only patients of the intervention group) will have access to a list where patient names and contact details can be associated with the pseudonyms. All study centres responsible for statistical analysis or monitoring of manual adherence will only receive pseudonymised data. All collected data will be stored within the safe firewall-protected network of the University of Freiburg.

#### Quality control of data

The research group will ensure data quality by following standard operating procedures (SOPs). The assessors will directly document LoChro-Care data in pseudonymised source data Case Report Forms (CRFs). Data will be checked for completeness, plausibility and incorrect data, which will lead to queries by the study site responsible for data management. If queries lead to data correction, this will be done by the assessors (source data CRFs) and the data management centre. The uniformity of corrections made at the data management centre will be checked during onsite monitoring visits. This procedure ensures source data verification. In order to ensure efficient and reliable monitoring and data management processes, the following SOPs of the “Technology, Methods, and Infrastructure for Networked Medical Research” (Technologie- und Methodenplattform für die vernetzte medizinische Forschung, TMF) will be adapted to specific requirements of the LoChro-Care study: (1) SOP monitoring visit: to ensure the reliable evaluation of identical corrections due to queries and the uniformity of data in the patient file at the study site and the data provided in the sociodemographic assessment. (2) SOP data check: to ensure identical procedures when incoming data are checked for correctness and queries are handled. (3) SOP data entry check: to ensure a rate of data entry errors of less than 0.2%. (4) SOP adverse event: to ensure that adverse events are disclosed early and handled appropriately.

#### Public dissemination, transfer and implementation

We plan five scientific publications, preferably in open access journals with high impact factors and international standards according to reporting guidelines (EQUATOR network): (1) study protocol; (2) the main paper reporting the complete study outcomes, no matter whether positive or negative; (3) a paper on the results of the process evaluation, which deepens the understanding of how the treatment might have worked and which potentially new hypotheses must be confirmed in further research; (4) a first version of a core outcome set in research on geriatric patients with chronic multimorbidity, which will be given consent by our “local” practice and scientific advisory board and should be further discussed in the international scientific literature and in practice and patient networks; (5) a first version of an intervention taxonomy, which defines and categorises the approaches applicable in stepped collaborative care management of older persons with chronic multimorbidity, as has been accomplished on a European level for fall prevention interventions.

Based on the appraisal of all care plans and modifications in the experimental group and based on profiles of existing local geriatric care services, we will develop a guideline on local best chronic care management. Furthermore, we plan to publish a user-friendly CCM manual. The members of our practice advisory board will be provided with user-oriented information material in plain language in order to disseminate the study results and the implications for practice via the groups they represent and their networks.

## Trial status

Enrolment for the trial began in February 2018. As of February 2019, recruitment and data collection is ongoing.

## Discussion

To our knowledge, this study is the first large-scale randomised controlled trial (RCT) to investigate cross-sectoral chronic care management for multimorbid older patients with functional impairment or chronic conditions. So far, only single-disease guidelines are available for the evidence-based management of depression [49], diabetes type 2 [50, 51], dementia [52] and frailty [53], even though these conditions are not only frequent in older people, but often also appear comorbidly. Guidelines to inform appropriate care for geriatric patients with multimorbidity, which address interactions of several diseases, disabilities, medication regimens and non-pharmacological intervention strategies, are lacking. Older persons with multimorbidity frequently experience fragmentation of care due to the involvement of a number of different care providers. In the present study, we will evaluate whether the urgent need to re-orient health service coordination and performance towards a stepped and collaborative care management approach, which is comprehensible, acceptable and effective, can be achieved by establishing chronic care managers and lay helpers in the health care provision of old and very old people and their family caregivers.

The inclusion and exclusion criteria define a study sample according to the complete spectrum of older persons with multimorbidity at risk of fragmented care management, with two exceptions. Accordingly, the findings cannot be generalised to persons who do not speak German and persons suffering from terminal conditions. We believe that it is justified from an ethical, scientific and economic perspective to first evaluate the effectiveness of LoChro-Care in the defined population. If substantial benefit can be found, further transfer studies with translators and additional specific end-of-life care management should be considered for implementation in these specific vulnerable older populations.

Positive results of the study would be a first step towards establishing new structures in the provision of health care for older patients with long-term health conditions. Based on the findings of this study, a local guideline on best regional chronic care management will be developed. Geriatric screenings at emergency departments could be implemented into routine care and CCM interventions introduced as usual care concepts. To support the implementation process, we plan to publish a user-friendly CCM manual and additionally, new CCM could be trained, also using the videos of the CCM sessions. If this study should find the chronic care intervention to be superior to usual care, further research on the implementation process would be necessary, which could consider findings of responders and non-responders to the intervention and thus optimise individual health care for older people with long-term health conditions.

## Abbreviations

AUDIT-C: Alcohol Use Disorders Identification Test-Consumption
BMBF: German Federal Ministry of Education and Research
CCM: Chronic Care Manager
COMET: Core Outcome Measures in Effectiveness Trials
CONSORT: Consolidated Standards of Reporting Trials
CRF: Case Report Form
DFG: German Research Foundation
DRKS: German Clinical Trials Register
EQUATOR-Network: Enhancing the Quality and Transparency of Health Research Network
FIMA: Fragebogen zur Erhebung von Gesundheitsleistungen im Alter
IADL: Instrumental Activities of Daily Living
ISAR: Identification of Seniors at Risk screening tool
JAMA: Journal of the American Medical Association
LoChro-Care: Local, collaborative, stepped and personalised care management for older people with chronic diseases
MMSE: Mini-Mental-State-Examination
PACIC: Patient Assessment of Chronic Illness Care
PCORI: Patient-Centered Outcomes Research Institute
PHQ-9: Patient Health Questionnaire
RCT: Randomised Controlled Trial
SOP: Standard Operating Procedures
SPPB: Short Physical Performance Battery
TMF: Technology, Methods, and Infrastructure for Networked Medical Research
TOPICS-MDS: The Older Persons and Informal Caregivers Survey Minimum Data Set
WHODAS: World Health Organization Disability Assessment Schedule 2.0
